# Multiscale Transcriptomic Integration Reveals B-Cell Depletion and T-Cell Mistrafficking in Nasopharyngeal Carcinoma Progression

**DOI:** 10.3389/fcell.2022.857137

**Published:** 2022-04-01

**Authors:** Xiaojie Shi, Junyan Pan, Fufang Qiu, Liqin Wu, Xuyan Zhang, Yan Feng, Xiaoyi Gu, Jikuang Zhao, Wenwei Zheng

**Affiliations:** ^1^ Department of Otolaryngology, Haining People’s Hospital, Jiaxing, China; ^2^ Department of Neurosurgery, Ningbo First Hospital, Ningbo Hospital of Zhejiang University, Ningbo, China; ^3^ Department of Pathology, Haining People’s Hospital, Jiaxing, China; ^4^ Department of Neurology, Haining People’s Hospital, Jiaxing, China

**Keywords:** transcriptomics, microenvironment, immunohistochemistry, nasopahryngeal carcinoma, bioinformatics

## Abstract

Nasopharyngeal carcinoma (NPC), featured by Epstein-Barr virus (EBV) infection and regional epidemiology, is curable when detected early, but highly lethal at an advanced stage. The molecular mechanism of NPC progression toward a clinically uncontrollable stage remains elusive. In this study, we developed a novel computational framework to conduct multiscale transcriptomic analysis during NPC progression. The framework consists of four modules enabling transcriptomic analyses spanning from single-cell, bulk, microenvironment, to cohort scales. The bulk-transcriptomic analysis of 133 NPC or normal samples unraveled leading functional enrichments of cell-cycle acceleration, epithelial-mesenchymal transition, and chemokine-modulated inflammatory response during NPC progression. The chemokine *CXCL10* in the NPC microenvironment, discovered by single-cell RNA sequencing data analysis, recruits cytotoxic T cells through interacting with its receptor *CXCR3* at early but late stages. This T-cell mistrafficking was featured by the decline of cytotoxic T cells and the increase of regulatory T cells, accompanied with B-cell depletion confirmed by immunohistochemistry staining. The featured immunomodulatory chemokines were commonly upregulated in the majority of cancers associated with viral or bacterial infections.

## Introduction

Nasopharyngeal carcinoma (NPC) is a special type of head and neck cancers, mostly associated with Epstein-Barr virus (EBV) infection. The worldwide incidence rate of NPC is lower than one per 100,000 persons, whereas the rate in certain regions is extremely high, including countries at southeastern Asia, north Africa and Alaska in the US (Chua et al., 2016; Tang et al., 2016; Chen et al., 2019). Remarkably, the incidence rate in Southern China ranges from 12.8 to 25 per 100,000, accounting for over a half of the total patients in the world (Tsang et al., 2020). This regional epidemic might be interpreted in part by a recent study demonstrating that the EBV isolates from China frequently carried two unique variants strongly associated with the regional infection (Xu et al., 2019).

Most low-grade NPCs are curable, whereas high-grade NPCs have significantly worse survival, and those with distant metastasis are often out of clinical control and extremely lethal (Li et al., 2014). The records at the American Joint Committee on Cancer (AJCC) between 2009 and 2015 have shown significant differences in the 5-year survival rates among different histological groups of NPC patients: 82% for low-grade primary tumors, 73% for high-grade with invasion at lymph nodes and peripheral tissues, and only 48% for those with distant metastasis. Therefore, understanding the underlying molecular and immunological features driving NPC progression is on high demand, which may help discover novel drug targets to prevent this deadly progression.

To understand the molecular landscape of NPC, several studies have used next-generation sequencing to explore genomic alterations (Lin et al., 2014; Li et al., 2017) and transcriptional dysregulation (Sengupta et al., 2006; Bose et al., 2009; Leek et al., 2012; Tang et al., 2018) between NPC tissues and health nasopharynx samples. Major molecular variation in NPC includes but not limited to chromosome 3p loss, *CDKN2A/B* loss in cell cycle control, and hyperactivation of NF-kB pathway driven by either loss-of-function somatic mutation of *CYLD* or overexpression of EBV oncoprotein *LMP1* (Tsang et al., 2020). In addition, through transcriptional comparison of 31 NPC samples and 10 healthy tissues, a previous study found that NPC tumor cells achieve immune evasion via EBV-mediated suppression of MHC-I type HLA genes (Sengupta et al., 2006). Recently, one study has identified 13 highly predictive biomarkers of metastatic risk of NPC, including *WSB2*, *FNDC3B* and *CXCL10*, and consolidated the clinical utility of these biomarkers in 937 patients in Southern China (Tang et al., 2018). The potential biological implication of these predictive biomarkers is worth further investigation.

In this study, we aim to investigate transcriptional and immunological signatures associated with NPC progression from low to high grades and distant metastasis. Through integration of four datasets of bulk microarrays comprising 116 NPC at different stages and 17 adjacent normal samples, we develop a novel computational framework based on statistical trend analysis to dissect immunological components in microenvironment and molecular hallmarks in NPC progression. Besides signatures of cell cycle acceleration, epithelial–mesenchymal transition and B-cell depletion, we discover an inflammation-associated signature, featured by the chemokine *CXCL10*, is in fact secreted by tumor-associated macrophages using single-cell RNA sequencing data. Finally, we demonstrate by independent pan-cancer omics datasets that these featured chemokines are pervasive in multiple viral-associated cancers.

## Methods

### Data Preparation and Integration

The microarray data of 116 NPC samples and 17 adjacent healthy nasopharynx samples used in the present study were downloaded from Gene Expression Omnibus (GEO) with accession code GSE12452 (Sengupta et al., 2006), GSE13597 (Bose et al., 2009), GSE64634 (Bo et al., 2015) and GSE103611 (Tang et al., 2018). The expression data were measured by different platforms and protocols and have strong batch effects that would affect downstream analysis. We therefore used *sva* package (Leek et al., 2012) to remove the batch effect, and then merged the four datasets by removing the genes that were not present in any of the four studies. Afterward, a quantile normalization was performed to avoid the influence of outlier data points. Principal component analysis (PCA) was performed to visualize at low dimensions whether the batch effect was removed.

For clinical information, we used the grades of lymph node spread (N) and diagnosis of overt metastasis (M) to split the tumors into three progression stages: Stage 1 includes the patients with N0M0 or N1M0; Stage 2 includes the patients with N2M0, N3M0, N0M1 or N1M1; and Stage 3 includes N2M1 or N3M1. The overall survival data from the dataset GSE64634 (Bo et al., 2015) were used to analyze survival difference among the three patient groups using *survival* package (Therneau, 2020), and was visualized using Kaplan-Meier plot (K-M plot).

### Statistical Trend Analysis

Given the stage variable *x* = 1, 2, 3, four denoting normal (Stage 0) and progression Stage 1, 2 and 3, respectively, and expression of each gene *y*, we estimated the trend of expression in different stages (*x*,*y*) in each sample using least squares fit $\sum {\left[y-\left(\beta_{1}x+\beta_{0}\right)\right]}^{2}$ . The null hypothesis is $\beta_{1}=0$ denoting no trend, and we performed a permutation test by shuffling the group variable 10,000 times at random to generate a Gaussian distribution centered at zero. An empirical *p* value was calculated to denote how likely the fitted $\beta_{1}$ is sampled from the distribution generated by the random shuffling. We selected the genes with *p* < 0.05 as the set associated with NPC progression. And the gene was sorted by the corresponding $\beta_{1}$ value and the ranking list was input into GSEA (Subramanian et al., 2005) under “pre-rank” mode to query enriched cancer hallmarks curated by the Molecular Signatures Database (MSigDB) (Liberzon et al., 2015).

### Single-Cell RNA-Sequencing Data Analysis

We downloaded the raw data of single-cell RNA sequencing of NPC samples at different progression stages from one previous study (Chen et al., 2020) under accession code CNP0000428 from the China National GeneBank Sequence Archive. The raw data were aligned and processed into count tables using CellRanger (Zheng et al., 2017) developed by 10x Genomics Inc. Further downstream analysis was performed using Seurat R package (Satija et al., 2015; Butler et al., 2018).

### Bulk Deconvolution of Bulk Expression Data

We used ConsensusTME (Jiménez-Sánchez et al., 2019) to computationally deconvolve the expression profiles of the 133 samples into 18 immunological components and one prognostic metric termed *immunoscore*. The parameters used in the ConsensusTME are “cancer = HNSC” and “statMethod = ssgsea”.

### Immunohistochemistry Staining of B Cells in NPC Samples

The formalin-fixed paraffin-embedded (FFPE) tissue slides were sectioned at 4 μm thick from the tumor specimens resected at Department of Otolaryngology, People’s Hospital of Haining, and were mounted on coated glass slides for immunohistochemistry (IHC) staining. The slides were stained by Ultra60-1,600 automatic IHC stainer (ZSGB-BIO, Beijing, China). Anti-CD20 antibodies (L26, dilution 1:2000, #ZM-0039, ZSGB-BIO, Beijing, China) were used for staining. Digital pathological images were scanned using digital whole slide scanning (HS6, 190,,011-6, SOPTOP, Ningbo, China), and were analyzed using the software Aperio-ImageScope (Leica, Version: 12.4.3). We obtained the digital images of the stained tissue sections at ×10 magnification.

### Pan-Cancer Analysis

We used Oncomine (Rhodes et al., 2007) to test whether inflammation-associated signature genes are significantly differentially expressed in multiple cancer transcriptomic datasets. Given one input gene symbol, Oncomine performed differential expression analyses using 715 datasets from the datasets of over 20 cancer types, and returned the number of analyses within which the input gene is significantly expressed between cancer and normal samples. In this study, the significant expression was defined as *p* value <0.001, log fold change >3 and *p*-value ascending ranking list of top 5% out of all the genes.

## Results

### Survival Difference at Different NPC Progression Stages

**FIGURE 1:**
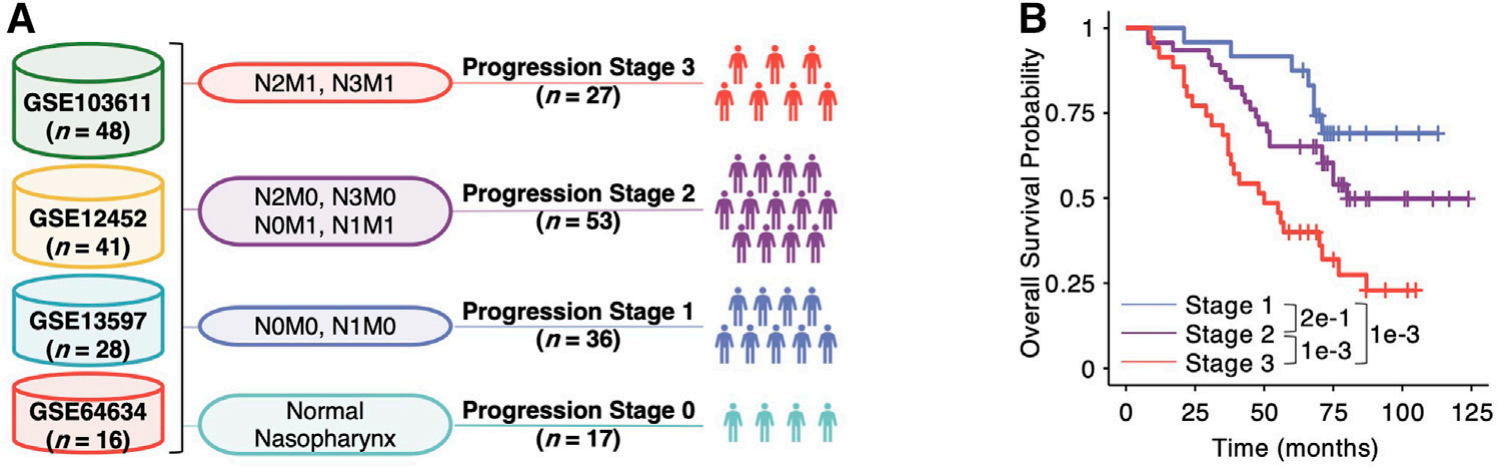
Analytic framework of multiscale transcriptomic integration for nasopharyngeal carcinoma (NPC) progression. **(A)** Schematic view of multi-center microarray data integration. The number *n* in the brackets denotes the sample size of each dataset. The lymph node spread (N0, N1, N2 and N3) and overt metastasis (M0 and M1) in the TNM system are used in the staging step. **(B)** Kaplan-Meier plot of NPC patients in Stage one, two and three of the integrated cohort. Pairwise *p* values between two different stages are derived from two-sided log-rank test.

To identify genes associated with NPC progression, we collected the microarray data of 133 NPC at different pathological grades and normal samples from four previous studies: GSE12452 (Sengupta et al., 2006), GSE13597 (Bose et al., 2009), GSE64634 (Bo et al., 2015) and GSE103611 (Tang et al., 2018). Using the measures of lymph node spread (N) and overt metastasis (M) in TNM system, we split the 133 samples into four progression stages (Figure 1A). To rationalize our strategy in progression staging, we used the strategy to split a cohort of 112 NPC patients with survival data available from GSE64634 (Bo et al., 2015) using the same criteria, which has shown to have different overall survivals (*p* = 0.21 between Stage 1 and 2, *p* = 0.0098 between Stage 2 and 3, and *p* = 0.001 between Stage 1 and 3, log-rank test, Figure 1B). Unexpectedly, we found that splitting the NPC patients using solely tumor size did not yield significant difference in overall survival (*p* = 0.38, log-rank test, Supplementary Figure 1A), and some patients with T4-tumors exhibit better survival than those with T1-tumors.

### Overview of Multiscale Analytical Framework

To enhance statistical power, we collected a large dataset of NPC progression by integrating the microarray expression data from the four studies and removing the batch effect generated by different sequencing protocols. Using Principal Component Analysis (PCA) to project all the 133 samples into a 2D dimensions, we showed a well-mixed distribution among different batches of expression data (Supplementary Figure 1B). In addition, we found that the expression profiles of NPC and normal nasopharynx samples are remarkably different, while the expression of NPCs at different stages are not well-separated (Supplementary Figure 1C). To further investigate the expression changes between different stages, we developed a multiscale analytic framework consisting of four modules (Supplementary Figure 1D): 1) trend analysis for comparison of different patients at cohort scale that captures key progression modulators with increasing average expression at sequential order of progression stages, 2) single-cell analysis for comparison of different molecules at single-cell scale which identifies crucial cell-cell communications via chemokine-receptor interactions, 3) bulk-deconvolution for comparison of different immune cell compositions at patient scale that consolidate significant changes of immune cell fractions observed at single-cell scale during NPC progression, and 4) pan-cancer analysis for comparison of different cohorts at population scale that investigate common aberrant gene expressions in multiple types of cancers.

### Transcriptional Signatures in NPC Progression

**FIGURE 2:**
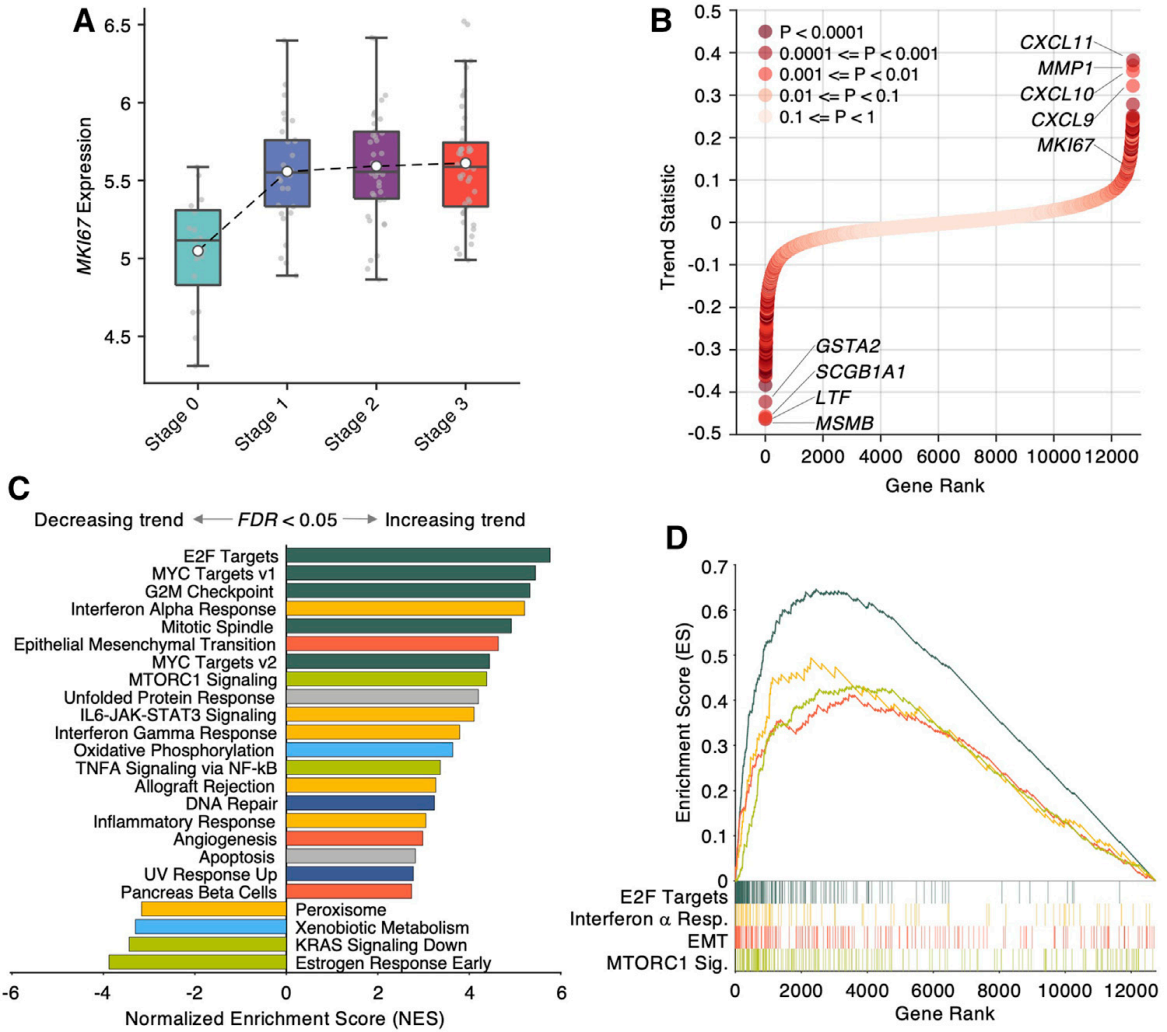
Trend analysis identifies transcriptional signatures in nasopharyngeal carcinoma progression. **(A)** Boxplot of *MKI67* expression in different NPC progression stages. **(B)** Scatter plot of trend statistic of each gene, sorted by ascending order. Colors denote trend significance, derived by 10,000 permutation tests. **(C)** Bar plot of Normalized Enriched Score (NES) derived from GSEA (Gene Set Enrichment Analysis) using 50 cancer-hallmark gene sets. The gene sets with FDR <0.05 are shown. Positive/negative NES denotes increasing/decreasing trend in NPC progression. Colors denote the functional category of each gene set. **(D)** Enriched score ranked by trend statistics. Four representative gene sets in different functional categories are shown.

To identify the expression patterns associated with NPC progression, we developed an empirical statistical method to test whether the expression of one gene exhibits a linear trend at progressive stage order (see Methods). Using the trend statistic beta and its empirical significance, we prioritized 12,746 genes based on their increasing/decreasing trends during NPC stage progression, and identified 2,698 genes with significant trends (*p* < 0.05). To demonstrate the rationale of the trend analysis, we showed the expression of *MKI67*, a prominent histological biomarker of cellular proliferation, in different stages and observed a remarkably increasing trend from Stage 0, a healthy control, to Stage 3, the final stage with distant metastasis (Figure 2A). Visualizing the trend statistics of all the genes in Figure 2B, we discovered that the genes with top increasing trends are *CXCL11*, *MMP1*, *CXCL10* and *CXCL9*, while the genes with the top decreasing trends are *MSMB*, *LTF*, *SCGB1A1* and *GSTA2*. Notably, *CXCL10*, a chemokine responsible for chemoattraction, has been demonstrated in an independent large-scale study as a significant prognostic biomarker of distant metastasis in locoregional advanced NPC patients (Tang et al., 2018).

To uncover biological insights of the prioritized genes by the trend analysis, we performed the Gene Set Enrichment Analysis (GSEA) using the trend statistic as a pre-rank metric. Remarkably, the GSEA revealed 24 significantly enriched cancer hallmarks (FDR <0.05, Figure 2C), among which the top functional hallmarks are mainly from four categories of biological processes: cellular proliferation (E2F targets, MYC targets v1 and v2, G2M checkpoint, Mitotic spindle, dark green bars in Figures 2C, D), immunity (interferon alpha, gamma responses, IL6-JAK-STAT3 signaling, and inflammatory response, yellow bars in Figures 2C, D), development (epithelial mesenchymal transition and angiogenesis, red bars in Figures 2C,D), and signaling (MTORC1 signaling and TNFA signaling via NFkB, light green bars in Figures 2C,D). Strikingly, epithelial mesenchymal transition (EMT) as a predominant phenotype of cancer metastasis, were found to be significantly associated with NPC progression (FDR <0.0001) with *MMP1* as the leading gene in the EMT gene set. In addition, another notable enriched hallmark is interferon gamma response (FDR <0.0001) with *CXCL9/10/11* as the leading genes in the set. These findings suggest that beside accelerated cell proliferation, NPC progression involved the activation of EMT program in which malignant nasopharyngeal epithelial cells undergo a morphological transition into a mesenchymal-like phenotype with strong invasive and migratory potentials, and the activation of immune response to recruit lymphocytes by upregulating *CXCL9/10/11*.

### Single-Cell RNA-Sequencing Analysis

**FIGURE 3:**
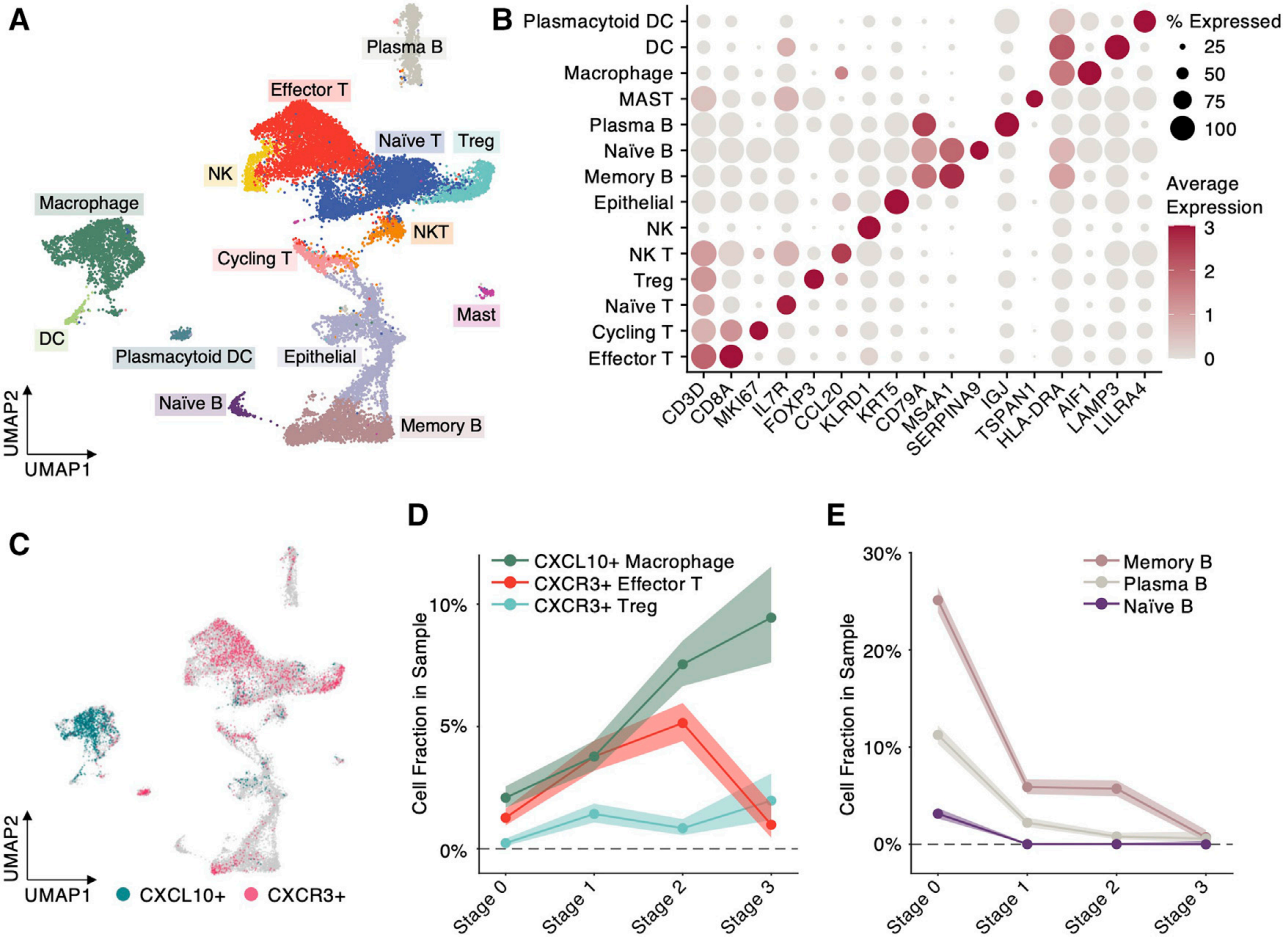
Single-cell RNA sequencing analysis uncovers cellular origin of progression-associated modulators. **(A)** Scatter plot of each cell at 2D UMAP space. Colors denote different cell types. **(B)** Scatter plot of average expression of biomarker genes in different cell types. Color intensity denotes expression level and dot size denotes percentage of cells expressing the biomarker. **(C)** Scatter plot of each cell at 2D UMAP space. Colors specify the cells highly expressing *CXCL10* or its receptor *CXCR3* (normalized expression >2). **(D)** Cell fractions, defined by the cell number highly expressing *CXCL10* or *CXCR3* divided by the total cell number in the cell type, at different NPC progression stages. Shaded area indicates 99% Confidence Interval. **(E)** B-cell fractions, defined by the B cell numbers divided by the total cell number in the sample, at different NPC progression stages. Shaded area indicates 99% Confidence Interval.

Evidenced by the trend analysis (Figure 2B), the functional enrichment (Figures 2C, D) and previous prognostic study (Tang et al., 2018), we inferred that the chemokine *CXCL10* might play a crucial role in tumor microenvironment of NPC progression. However, its biological role and dynamics in NPC progression remain largely unknown. To first investigate the cellular origin of *CXCL10* in NPC progression, we collected single-cell RNA sequencing (scRNA-seq) data from a recent study with NPC patients at different stages (Chen et al., 2020). From the scRNA-seq dataset, we selected and integrated four samples from normal nasopharynx and NPC tumors at Stage 1, 2 and 3, respectively. The integrated expression profiles of 20,841 cells were projected into a 2D space (Figure 3A) using UMAP by Seurat package, and were further categorized into 14 cell types based on regular cell markers (Figure 3B). Similar to the microenvironment of other cancer types (Puram et al., 2017; Zhang et al., 2020), the NPC microenvironment consists primarily of T cells, B cells and myeloid cells (Figure 3A).

Highlighting the cells with highly expressed *CXCL10* and its binding receptor *CXCR3*, we uncovered that *CXCL10* is predominantly expressed by macrophages and its receptor *CXCR3* is mainly expressed by T cells, indicating macrophages attract T cells through *CXCL10*-*CXCR3* communication (Figure 3C). Notably, the cell proportion of *CXCL10* + macrophages exhibit a significantly increase (*z*-test, Supplementary Figure 2A) from Stage 0 (normal) toward Stage 3 (metastasis) during NPC progression (Figure 3D), which is consistent with the trend identified by the trend analysis (Figure 2B). However, the *CXCL10* + macrophages do not keep recruiting *CXCR3*+ effector T cells: the proportion of *CXCR3*+ effector T cells dramatically declines from 5.14% at Stage 2 to 0.98% at Stage 3 (*z*-test, Figure 3D and Supplementary Figure 2A). In contrast, the cell proportion of *CXCR3*+ regulatory T cells (Treg), which play a suppressive role in immune response, shows a significantly ascending trend from 0.84% at Stage 2 toward 1.97% at Stage 3 (*z*-test, Figure 3D and Supplementary Figure 2A). These results imply that macrophages recruit *CXCR3*+ effector T cells through *CXCL10* expression at early stage of NPC progression, but at late stage, the NPC microenvironment might undergo a reprogramming from an active into a suppressive state, evidenced by the decline of *CXCR3*+ effector T cells and the increase of *CXCR3*+ regulatory T cells. In addition, we observed significant depletion of B cells during NPC progression (*z*-test, Figure 3E and Supplementary Figure 2B). Especially, memory B cells dramatically drop from 25.11% at Stage 0–0.72% at Stage 3, which suggests that like EBV-associated B-cell lymphomas, the B cells in NPC are vulnerable under EBV infection.

### Deconvolved Immunological Composition and Biomarkers in NPC Progression

**FIGURE 4:**
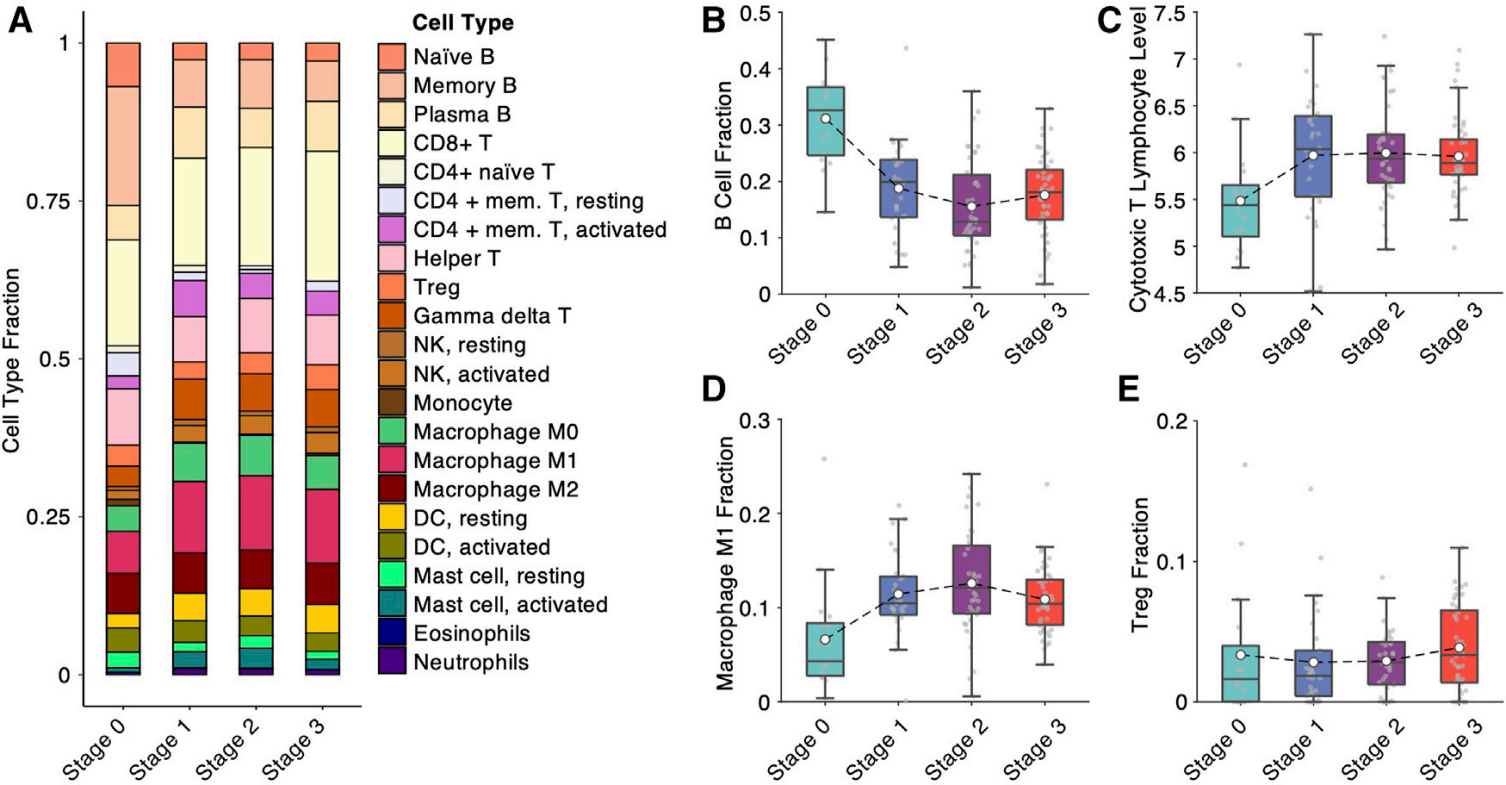
Bulk deconvolution analysis reveals immunological variation during nasopharyngeal carcinoma progression. **(A)** Cell-type fractions deconvolved from bulk expression data measured by microarray from 113 samples in the integrated cohort. **(B)** B-cell fraction from bulk deconvolution at different NPC progression stages. **(C)** Cytotoxic T lymphocyte level, estimated by average expression of *CD8A*, *CD8B*, *GZMA*, *GZMB* and *PRF1*, at different NPC progression stages. **(D)** M1 macrophage fraction from bulk deconvolution at different NPC progression stages. **(E)** Treg (regulatory T) cell fraction from bulk deconvolution at different NPC progression stages.

To further validate the dynamic changes of NPC microenvironment during progression discovered by the scRNA-seq analysis, we computationally estimated 22 immune cell proportions in the bulk microarray data of 133 NPC and normal samples (Figure 4A) using ConsensusTME (Jiménez-Sánchez et al., 2019). One of the apparent changes among the 22 immune cells is B cell depletion during the progression (Figure 4B), which is consistent with the observation of scRNA-seq analysis (Figure 3E). Estimating the cytotoxic T lymphocyte (CTL) level of each sample using the average expression of five marker genes (*CD8A*, *CD8B*, *GZMA*, *GZMB* and *PRF1*) (Rooney et al., 2015), we showed that the CTL levels boost up at the early stage but slightly drop at the late stage during NPC progression (Figure 4C). Similarly, the proportion of anti-tumor M1 macrophages display a trend of “increase first, decrease later” (Figure 4D). Consistent with the scRNA-seq analysis (Figure 3D), we also observed an increasing trend of Treg cell proportion in the deconvolution analysis (Figure 4E). All the evidence of the analyses pointed to a unique trend of tumor microenvironment during NPC progression: from active immune surveillance at the early stage to immunosuppressive’ at the late stage.

### Validation of B-Cell Depletion via Immunohistochemistry Staining

**FIGURE 5:**
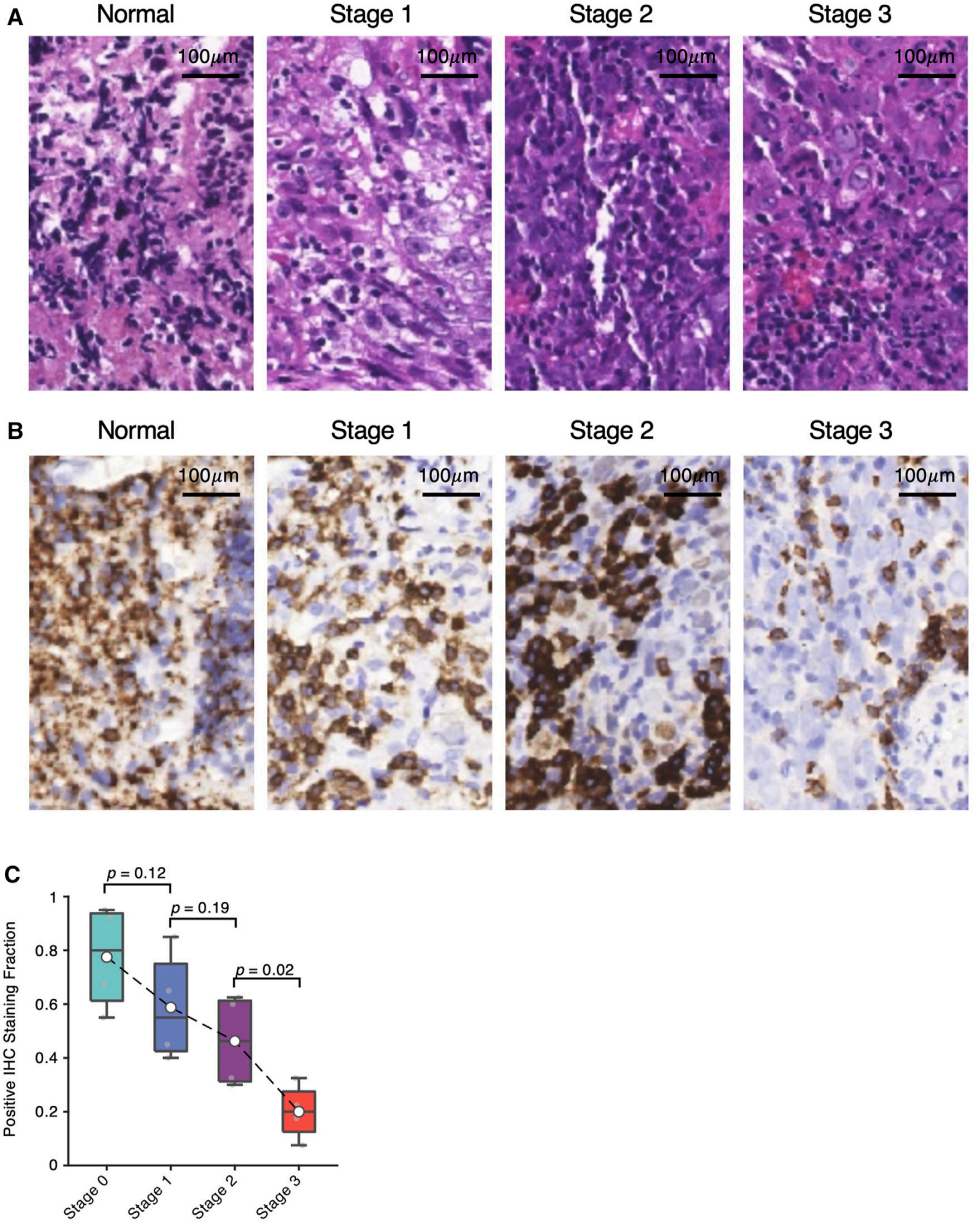
Validation of B-cell depletion via immunohistochemistry staining. **(A)** Hematoxylin and eosin (HE) staining images of Stage one, two and three NPCs and non-NPC adjacent normal samples. **(B)** Corresponding anti-CD20 immunohistochemistry (IHC) staining images of Stage one, two and three NPCs and non-NPC adjacent normal samples. **(C)** Fraction of IHC positive staining shown in **(B)** with significance estimated by *t*-test.

Enlightened by the consistent trends of B-cell depletion observed in the scRNA-seq and bulk deconvolution analyses, we conducted hematoxylin and eosin (HE) staining (Figure 5A) and anti-CD20 immunohistochemistry staining (Figure 5B) of three NPC samples at Stage 1, 2 and 3, and one adjacent normal sample collected by the Department of Otolaryngology, People’s Hospital of Haining, Zhejiang, China. Consistently, we observed a decreasing trend of the number of CD20-positive cells from Stage 1 to Stage 3 NPC samples (Figure 5C), which consolidates the computational results of B-cell depletion during NPC progression.

### Chemokine Upregulation at Pan-Cancer Scale

Commonly, the chemokines *CXCL9*, *CXCL10* and *CXCL11* are thought to be induced by interferon (IFN)-γ against inflammation (Metzemaekers et al., 2017). Using Oncomine (Rhodes et al., 2007), we investigated what types of cancers are associated with the upregulation of the three chemokines observed in NPC progression. Consistent with the observation in NPC, we found that the three chemokines are upregulated in 11 transcriptomic studies of head and neck cancer (Supplementary Figure 3A). Interestingly, the cancers in which the three chemokines are upregulated are significantly enriched with viral or bacterial infections (*p* < 0.05, *z*-test, Supplementary Figure 3A). Within the 10 cancers with the chemokine upregulation, six cancers (lymphoma, head and neck cancer, liver cancer, colorectal cancer, cervical cancer and bladder cancer) are associated with viral or bacterial infection. In contrast, only one cancer (gastric cancer) out of the other 10 cancers without chemokine upregulation is associated with bacterial infection. This suggests that the upregulation of *CXCL9*, *CXCL10* and *CXCL11* is a common event in the cancers with viral or bacterial association.

## Discussion

To investigate the dynamic patterns of NPC transcriptomes in progression, we developed a computational model to perform multiscale transcriptomic analysis. The entire analytic framework spans from the single-cell RNA-sequencing analysis at cellular scale, the trend analysis at molecular scale, the deconvolution analysis at immunological-component scale, and the pan-cancer analysis at cohort scale. Using this multiscale analytic pipeline, we have discovered that the expression trend during NPC progression is significantly enriched in the biological processes of cell-cycle acceleration, elevated invasiveness, and chemokine-modulated inflammatory response. In particular, the leading chemokine is *CXCL10* that interacts with its receptor *CXCR3* to modulate cytotoxic T cell trafficking from high to low levels during NPC progression. Pan-cancer analysis independently verified the prevalence of *CXCL10* upregulation in other cancers with viral or bacterial association. In addition, B cell depletion is another hallmark event during NPC progression. Taken together, our multiscale transcriptomic analytic framework pinpointed *CXCL10* as a crucial immune modulator in NPC microenvironment during progression.

## Data Availability

The original contributions presented in the study are included in the article/Supplementary Material further inquiries can be directed to the corresponding authors.
