# Supplementary material for: Multiscale Transcriptomic Integration Reveals B-Cell Depletion and T-Cell Mistrafficking in Nasopharyngeal Carcinoma Progression
Source: Front Cell Dev Biol. 2022 Apr 1;10:857137. doi: 10.3389/fcell.2022.857137 (PMC9011158; doi:10.3389/fcell.2022.857137)
Supplement: Supplementary file 1 [file DataSheet1.pdf]

Supplementary Figure 1

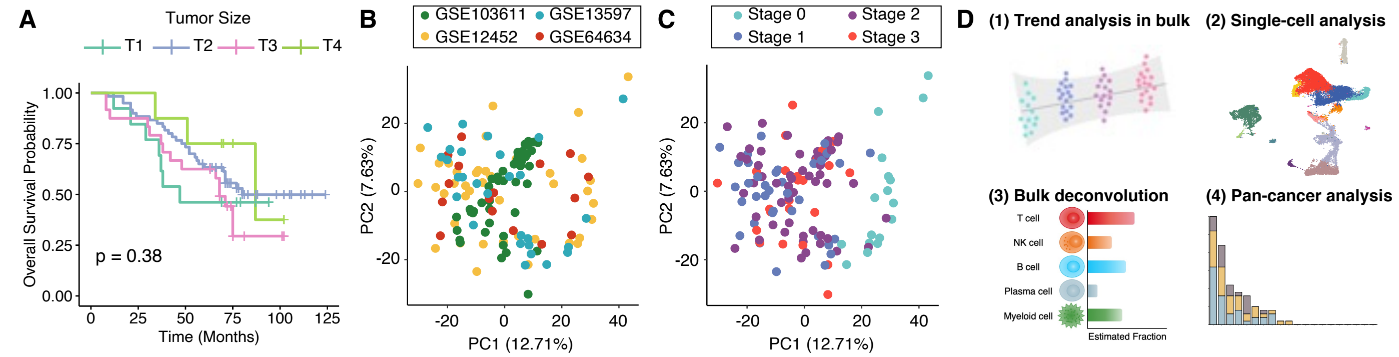

Supplementary Figure 1. (A) Kaplan-Meier plot of NPC patients in different tumor sizes denoted by TNM system classification. *P* value is derived from two-sided log-rank test. **(B)** and **(C)** Scatter plot of expression profile projected by PCA in 2D space. Each dot denotes the projection of one microarray sample. Percentages in the brackets denote the variance explained by the PCA projected dimension. Colors in **(B)** denote different datasets annotated by corresponding GSE ID, and colors in **(C)** denote different progression stages. **(D)** Four-step workflow of multiscale transcriptomic analysis for NPC progression.

Supplementary Figure 2

A

CXCR3+ Treg				
p-value	Stage_0	Stage_1	Stage_2	Stage_3
Stage_0		2.66E-15	1.08E-06	0
Stage_1			0.002925	0.1203
Stage_2				1.77E-04
Stage_3				

CXCR3+ effector T				
p-value	Stage_0	Stage_1	Stage_2	Stage_3
Stage_0		0.00E+00	0.00E+00	0.357759
Stage_1			0.000268	3.18E-08
Stage_2				1.02E-12
Stage_3				

CXCL10+ macrophage				
p-value	Stage_0	Stage_1	Stage_2	Stage_3
Stage_0		3.20E-09	0.00E+00	0
Stage_1			0	0
Stage_2				1.49E-02
Stage_3				

B

plasma B				
p-value	Stage_0	Stage_1	Stage_2	Stage_3
Stage_0		0	0	0
Stage_1			5.39E-10	2.81E-05
Stage_2				0.392912
Stage_3				

memory B				
p-value	Stage_0	Stage_1	Stage_2	Stage_3
Stage_0		0	0	0
Stage_1			0.708872	0
Stage_2				2.22E-16
Stage_3				

naïve B				
p-value	Stage_0	Stage_1	Stage_2	Stage_3
Stage_0		0	0	2.20E-12
Stage_1			0.900347	0.628768
Stage_2				0.597386
Stage_3				

Supplementary Figure 2. (A) Corresponding pairwise P values of cell fraction between different stages shown in **Figure 3D**. **(B)** Corresponding pairwise P values of cell fraction between different stages shown in **Figure 3E**.

Supplementary Figure 3

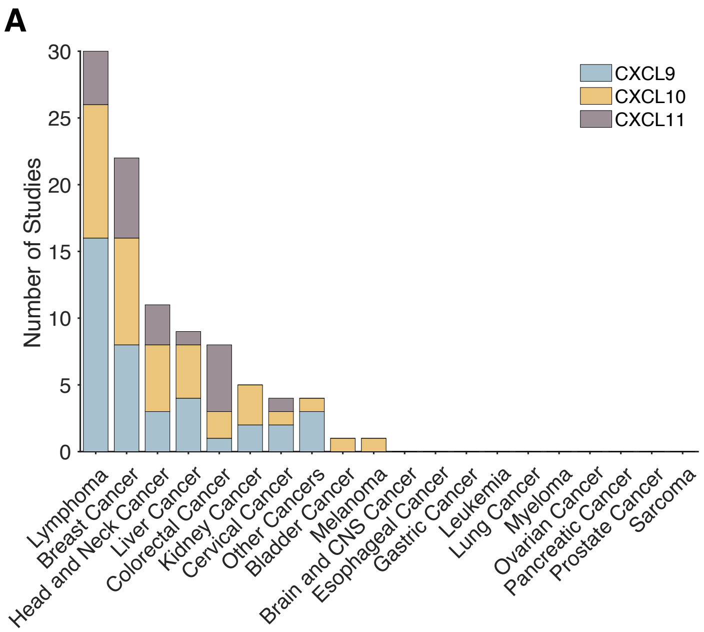

Supplementary Figure 3. Pan-cancer analysis consolidates featured chemokine upregulation with viral association. (A) Bar plot of number of studies supporting significant upregulation of chemokines in their expression data. Significant upregulation is defined as *P* value < 0.001, log fold change > 3 and *P*-value ascending ranking at top 5% of all the genes.
